# A case of primary biliary cirrhosis associated with pernicious anemia: a case report

**DOI:** 10.1186/1757-1626-3-11

**Published:** 2010-01-08

**Authors:** Elhem Ben Jazia, Mabrouk Khalifa, Atef Ben Abdelkader, Naoufel Kaabia, Neirouz Ghannouchi, Ahlem Braham, Amel Letaief, Fethi Bahri

**Affiliations:** 1 Department of Internal Medicine and Infectious diseases, University Hospital F Hached,4000, Sousse, Tunisia; 2 Department of Anatomical Pathology, University Hospital F Hached, 4000, Sousse, Tunisia

## Abstract

Primary biliary cirrhosis is often associated with autoimmune diseases. However, its association with pernicious anemia has rarely been reported.

We report a case of a 68-year-old woman who presented jaundice and pruritus. Mildly elevated serum levels of alkaline phosphatase and γ-glutamyl transpeptidase were detected. The titer of anti-mitochondrial M_2 _anti-body was elevated. Histology of liver biopsy showed features of primary biliary cirrhosis. In addition, aregenerative macrocytic anemia was found in the full blood count. The diagnosis of pernicious anemia was established by megaloblastosis in bone marrow, atrophic gastritis without Helicobacter pylori, low level of vitamin B_12 _and good response to treatment regimen of vitamin B_12_. The association of primary biliary cirrhosis and pernicious anemia is unlikely to be casual and may be explained by autoimmune mechanism commonly shared by the diseases.

## Introduction

Primary biliary cirrhosis (PBC) is a chronic autoimmune liver disease of unknown aetiology. It's characterized histologically by chronic non suppurative destruction of interlobular bile ducts leading to advanced fibrosis, cirrhosis and liver failure [1].

This disease may be associated with various autoimmune disorders, and a link with pernicous anemia (PA) has seldom been described [2].

## Case report

A 68-year-old woman was admitted in December 1999 to our department for jaundice, fatigue and pruritus. Her previous medical history was unremarkable. There was no history of alcohol, drug abuse, or family history of liver disease. Physical examination revealed hepatomegaly and splenomegaly. Her skin and sclerae were icteric. There was no cutaneous xanthoma and no spider angioma. Hepatic laboratory investigations found a cholestatic alkaline phosphatase: 4950 UI/l (N: 40 UI/l), γ-glutamyl transpeptidase: 108 IU/l (N < 50 IU/l), direct bilirubin level: 75 μmol/l (N < 5 μmol/l). The serum concentration of total cholesterol, albumin, and immunoglobulin, especially IgM level were within the normal range, the prothrombin time was normal.

The full blood count revealed macrocytic aregenerative anemia; hemoglobin 10 g/dl, mean corpuscular volume (MCV) value of 124 fl, absolute reticulocyte was 60 × 10^3^/mm^3^. Serum vitamin B_12 _level was decreased at 60 pg/ml. Viral serologic tests of hepatitis B and C were both negative. Antinuclear antibodies and anti smooth muscle antibody were negative. Whereas anti mitochondrial antibody was positive at 1/400 by immunofluorescence. Furthermore, anti parietal cell antibody (PCA) and anti intrinsic factor antibody were negative. Abdominal ultrasound examination showed hepato-splenomegaly without other signs of portal hypertension and excluded extra hepatic biliary obstruction.

**Figure 1:**
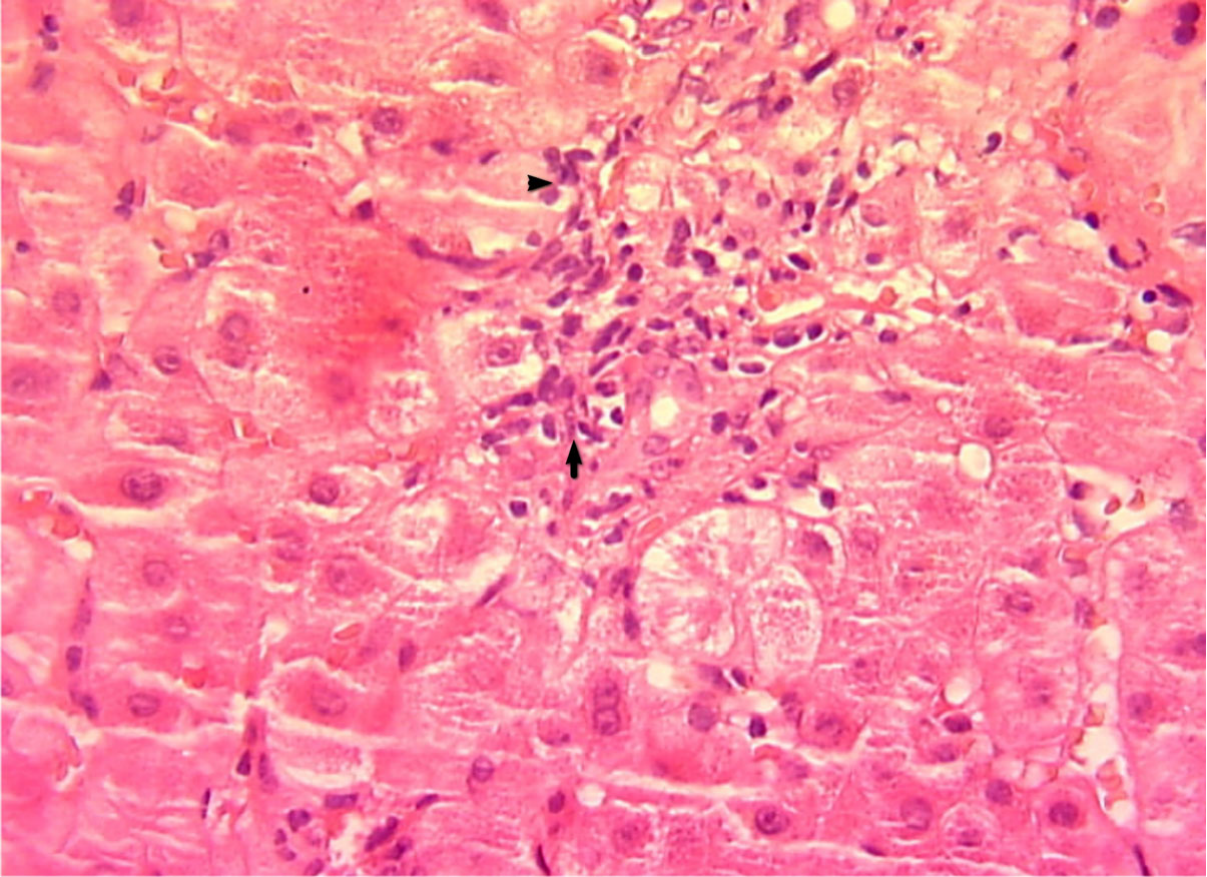
**HE stain × 400: portal infiltrate composed of lymphocytes invading the bile duct epithelium (Arrow) and extension to the peri portal areas (Head of arrow)**.

Histological examination of a liver biopsy revealed a portal inflammatory infiltrate mainly composed of lymphocytes invading and destroying the epithelium of bile ducts. Inflammatory cells also extend to the peri-portal areas (Fig. 1). Elsewhere, there was fibrosis peri-portal without regenerative nodules. The bone marrow biopsy showed megaloblastosis. Upper gastro intestinal endoscopy exhibited an atrophy of the gastric mucosa in the body of the stomach. Gastric biopsy specimens showed chronic inflammatory infiltrate into the lamina propria associated with destruction of parietal cells and foci of intestinal metaplasia and glandular atrophy. No Helicobacter pylori was seen

The diagnosis of PBC and Pernicious anemia was made. The patient was treated by ursodeoxycholic acid (UDCA = 800 mg/day) and intramuscular injection of vitamin B_12_. After 3 months of treatment, a significant improvement of laboratory data was established. In fact, hemoglobin levels increased to 11,8 g/dl and MCV decreased to 86 fl. Serum bilirubin gradually decreased, alkaline phosphatase and γ-glutamyl transpeptidase were normalized. Biliary enzyme, albumin concentration and prothrombin time remained within the normal range during the following four years under UDCA and Vitamin B_12_. Her hemoglobin rose to 13 g/dl.

## Discussion

PBC is a rare chronic cholestatic liver disease most often diagnosed in middle-aged women. It is characterized by a destruction of the bile ducts, portal inflammation causing hepatocyte destruction and extensive fibrosis. Ultimately, liver cirrhosis and liver failure ensue [1].

PBC is quite rare in Tunisia, the prevalence and incidence is unclear, because few epidemiologic studies have been established. In our patient, the diagnosis of PBC was confirmed on the basis of laboratory findings, positive anti mitochondrial antibody and histopathological findings compatible with PBC on liver biopsy.

PBC is well known to be associated with auto immune disorders including Sjögren's syndrome, rheumatoid arthritis, chronic thyroiditis and scleroderma. However, few cases of PBC associated with PA have been reported. The different cases reported in the literature are represented in table 1.

**Table 1:** Characteristic of cases reported in the literature about association of primary biliary cirrhosis and pernicious anemia

	Aoyama H	Renoux M			
**Number of case**	**1**	**Obs.1**	**Obs.2**	**Obs.3**	**Obs.4**
reference	2	7			
Age	59	68	46	66	72
gender	woman	woman	woman	woman	woman
Hepatic laboratory	cholestasis	cholestasis	cholestasis	cholestasis	cholestasis
Anti-mitochondrial antibody	positive	positive	positive	positive	positive
Histological finding of liver biopsy	Espanded portal areas with fibrosis and the scanty bile ducts	Extended fibrosis portal and ductopenia	Extended fibrosis portal and ductopenia	Extended fibrosis portal	No mentioned
Macrocytic anemia	yes	yes	yes	yes	yes
Serum vitamin B_12_	low	low	low	low	low
Anti-parietal cell antibody	positive	negative	-	negative	-

Pernicious anemia is the end stage of atrophic gastritis (type A gastritis) which results in the loss of parietal cells in the fundus and body of the stomach. Loss of parietal cells is associated with the failure of intrinsic factor production and results in vitamin B_12 _deficiency and megaloblastic anemia [3]. The presence of mononuclear cell infiltration into the gastric mucosa, parietal cell antibody (PCA) and anti-intrinsic factor antibody were in favour of the autoimmune basis for the gastritis [2]. Although, they were negative in our patient, the diagnosis of PA was established by macrocytic anemia in peripheral blood, megaloblastosis in bone marrow, atrophic gastritis without Helicobacter pylori, low serum vitamin B_12 _concentration and good response to treatment using vitamin B_12_.

Some authors have reported that PCA are frequently detected in patients with PBC[4] Oya et al. demonstrated that severe and extensive gastric mucosal atrophy was manifested in patient with PBC, which exhibited positive PCA [4]. On the other hand, Wirth reported that none of the patients with PBC and PCA had associated PA. Moreover, Floreani demonstrated that the prevalence of chronic atrophic gastritis is similar in PBC and dyspeptic controls [5]. This data supported that the presence of atrophic gastritis in PBC remains a controversial subject. Tissue damage in patients with PBC may be present in the salivary glands and lacrymal glands, as well as other exocrine glands. This is known as dry glands syndrome which results from damage to the ductular epithelia by a common autoimmune mechanism. The stomach also has an exocrine glandular structure. It's possible that atrophic gastritis is part of the dry-gland syndrome [6,7].

In conclusion, although PBC may occur in patients affected by another immuno-mediated disorder, its coexistence with PA is not frequently described. This association does not seem casual and may be pathogenically explained by autoimmune mechanism that they have in common.

## Consent

Written informed consent was obtained from the patient for publication of this case report and accompanying images. A copy of the written consent is available for review by the Editor-in-Chief of this journal.

## Competing interests

The authors declare that they have no competing interests.

## Authors' contributions

EBJ, MK: contributed equally to this work

ABA, NK: designed research

NG, Ahlem Braham, AL, FB: performed research

All authors read and approved the final manuscript.
